# Combination of dabrafenib and radiotherapy: could skin toxicity be affected by different irradiation techniques?

**DOI:** 10.1259/bjrcr.20150493

**Published:** 2016-05-08

**Authors:** Daniela Alterio, Giulia Marvaso, Annamaria Ferrari, Ombretta Alessandro, Emilia Cocorocchio, Pier Francesco Ferrucci, Roberto Orecchia, Barbara Alicja Jereczek-Fossa

**Affiliations:** ^1^Department of Radiation Oncology, European Institute of Oncology, Milano, Italy; ^2^Department of Oncology and Hemato-oncology, University of Milan, Milano, Italy; ^3^Medical Oncology of Melanoma, European Institute of Oncology, Milano, Italy

## Abstract

The prognosis of patients with metastatic melanoma is changing owing to the introduction of selective BRAF inhibitors combined with MEK inhibitors. Management of these patients continues to be a challenge, especially when systemic therapy has to be combined with concomitant radiotherapy, particularly owing to skin toxicity. Here we report a case of a patient who underwent concomitant treatment for two vertebral sites using two different radiotherapy techniques. An unexpected acute skin toxicity was reported at one of the treated sites. This finding might be owing to the different absorbed dose to the subcutaneous tissues linked with the technique of irradiation.

## Background

Melanoma remains the most aggressive skin cancer.^1^ Distant metastases often develop during the course of melanoma and are considered responsible for the death of half of the patients.^2^ The development of new drugs such as the selective BRAF inhibitors (BRAFi) for monotherapy or in combination with MEK inhibitors (MEKi) has revolutionized the treatment of metastatic BRAF V600-mutated advanced stage melanoma.^3–5^ Along with the systemic approach, target-directed radiation therapy (RT) is frequently required for symptomatic treatment in the management of these patients.

Preclinical studies suggested that target agents used to inhibit the *BRAF* gene can enhance the radiosensitivity of the tissues.^6^ Consequently, the concomitant administration of BRAFi (vemurafenib or dabrafenib) and RT may have a synergistic effect, with a potential increase in RT-related side effects. Many studies have reported different skin, lung and bowel toxicities in patients treated with both radiation and BRAFi.^7,8^ The degree and length of the duration of these toxicities are variable and not well defined. Moreover, while the skin toxicity side effects (including rash, photosensitivity and secondary skin malignancies) associated with the concomitant administration of vemurafenib and RT are widely reported,^9,10^ few cases of radiodermatitis owing to the combined use of dabrafenib and RT were found in the literature.^11^

The underlying mechanisms of these combined toxicities are yet unclear. Given the recent evidence suggesting that both dabrafenib and vemurafenib have shown unexpected side effects when used concomitantly with RT, the approach of this combined treatment is quite variable and there are no shared guidelines. The main concern is the interruption of the systemic therapy while the patients are undergoing RT, as this interruption can lead to a disease progression or delay in relief of symptoms.

Here we report a case of a patient with metastatic melanoma treated with dabrafenib and concomitant RT for vertebral bone metastasis. The radiation treatment was administered using two different three-dimensional (3D) conformal RT techniques for two different vertebral sites. The patient experienced differentiated radiation-related in-field skin toxicity related to the different RT doses to the subcutaneous tissue.

## Case presentation

In April 2015, a 58-year-old male with a history of recurrent melanoma, currently Stage IV, was admitted to our RT department for back pain owing to bone lesions at the T10–T12 vertebral levels. No peripheral neurological symptoms were present but vertebral lesions were at a high risk of fracture with consequent spine compression. In February 2009, he was diagnosed with two nodular melanomas in his back, which were treated with local excision. Thereafter, he remained free of disease until March 2015. At that time, he underwent CT/positron emission tomography scans for persistent pain in the lower back region with impaired deambulation, which was treated using anti-inflammatory drugs with no clinical benefit. The CT/positron emission tomography scans showed multiple metastatic lesions (brain, bone, lymph nodes and skin). Biopsy from a skin metastasis site revealed a BRAF V600E-mutated melanoma. Therefore, systemic therapy with dabrafenib was started at a standard dose (150 mg twice daily) while it was planned to start the MEKi (trametinib) after 2 weeks within the expanded access program. In our patient, trametinib was administrated about 5 weeks after the end of radiation course.

**Figure 1.**
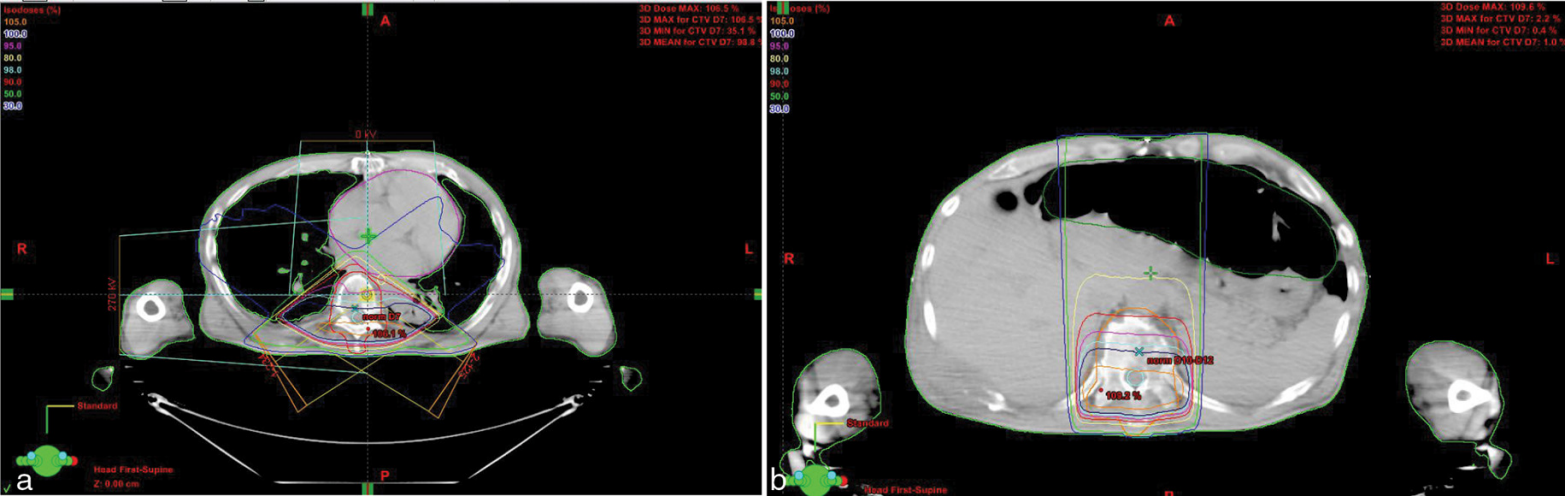
The CT-based plan of three-dimensional conformal radiotherapy using a linear accelerator with 18 MV photon beam. The treatment of T7 was performed using two oblique posterior–anterior fields (a) while the treatment for T10–T12 was performed using a direct posteroanterior field (b). The target volume was contoured by the red line in (a) and orange line in (b). The red and yellow isodoses encompass the area covered by 90% and 80% of the prescribed dose, respectively.

**Figure 2. (a, b):**
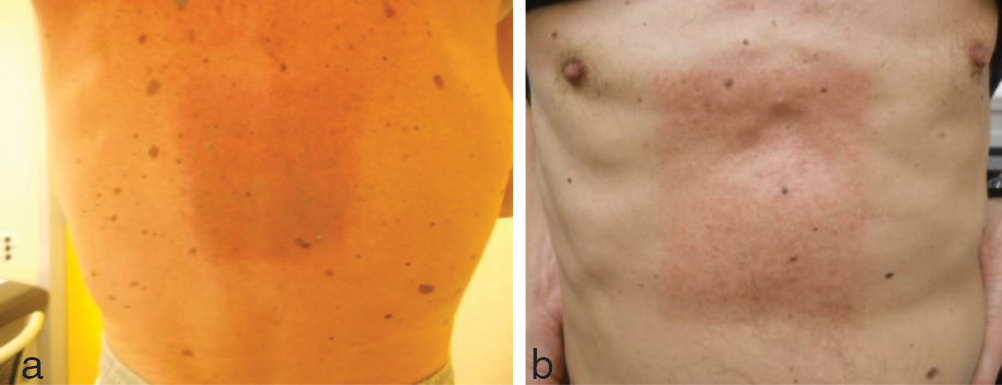
T10–T12 in-field radiation-related acute skin toxicity.

**Figure 3.**
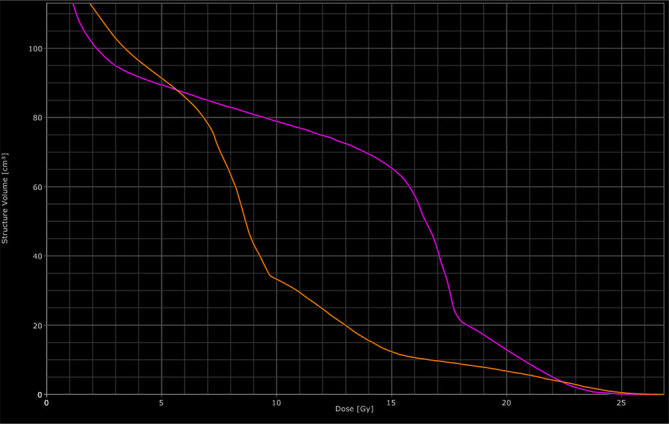
Dose volume histogram. The purple line represents the absorbed dose by the subcutaneous tissues of the T10–T12 field. The orange line represents the absorbed dose by the subcutaneous tissues of the T7 field.

For his bone lesions (T10–T12 and T7 vertebrae), the patient was soon scheduled for RT at a dose of 30 Gy administered in 10 fractions (3 Gy per fraction for 5 days a week). Because of a rapidly evolving disease, which needed a rapid and hopefully consistent response, dabrafenib was not interrupted during RT. Two different 3D conformal RT techniques were used: an isocentre technique with two oblique wedge pair fields for the T7 lesion and a direct skin–source distance posteroanterior field for the T10–T12 vertebrae using an 18 MV linear accelerator (Figure 1). After six fractions of RT (18 Gy), an increasing, unexpected skin toxicity appeared in the field of irradiation at the T10–12 level, both on the back and the abdominal region (Figure 2). This acute side effect was classified as Grade 2 radiodermatitis [according to the Common Terminology Criteria for Adverse Events (CTCAE) version 4.0].^11^ No acute skin toxicity or other systemic toxicity were documented in the field of the T7 vertebra. To further understand why the skin toxicity occurred in only one of the irradiated fields, the dose distribution of the two different RT treatment plans was reviewed. The absorbed doses to the target volumes (90% of the volume absorbed 95% of the prescribed dose for both volumes) and the maximum dose were found to be similar for the two plans. On the contrary, the volume of subcutaneous tissues that received a high dose was found to be significantly larger for the T10–T12 field than for the T7 field. In particular, the mean doses, V10, V15 and V20 (volume that absorbed 10, 15 and 20 Gy, respectively) were 78 and 33 cm^3^, 65 and 12 cm^3^ and 13 and 7 cm^3^, respectively, for T10–T12 and T7 (Figure 3). Moreover, 50 cm^3^ of subcutaneous tissues absorbed 16.5 and 8.7 Gy for the T10–T12 and T7 field, respectively. The subcutaneous tissues of lateral and anterior chest wall absorbed a mean and maximum dose of 14 and 18 Gy, and 7.5 and 8 Gy for T10–T12 and T7, respectively, confirming that the exit dose washigher for the T10–T12 than the T7 field. Owing to this toxicity, after a multidisciplinary discussion, the radiation course was stopped at a total dose of 18 Gy for both the irradiated volumes. At the 3-month follow-up, the patient had a significant pain reduction without the appearance of neurological symptoms and a new CT scan revealed a stable osseous disease.

From the clinical point of view, these findings open the discussion to whether the acute skin toxicity caused by the treatment with BRAFi in association with RT should be prevented by reducing high dose areas to the skin and the subcutaneous tissues.

## Conclusions

The literature about acute toxicity owing to the use of RT together with BRAFi is limited, and heterogeneous in terms of RT procedures (including conventional and stereotactic RT) and regarding the prevalent management of melanoma brain metastases. These data do not permit any definitive conclusions to be drawn.

According to Pulvirenti et al,^12^ the interaction of BRAFi with ionizing irradiation leads to further activation, repopulation and proliferation of normal tissue keratinocytes (wild-type), thereby facilitating more intense radiation-induced skin reactions.^12^

Behind the suggested biological hypothesis looking at the phenomenon from a strict RT point of view, the RT technique seems to play a crucial role in the pathogenesis of skin toxicity. Most of the published studies reporting skin toxicity refer to RT for brain metastases and the association with BRAFi. No or less skin toxicity was reported when stereotactic brain irradiation was applied; on the other hand, the use of whole-brain irradiation was correlated with higher incidence of radiation-related skin side effects. On the contrary, a higher incidence of tumour haemorrhage was described in patients treated with a stereotactic technique. It must be kept in mind that, from a technical point of view, stereotactic RT is different from the 3D conventional RT modality. The stereotactic technique uses multiple fields, concentrating the high dose to a small volume to ensure that the maximum dose is delivered to the target volume, while sparing the surrounding normal healthy tissue. Similarly, intensity-modulated radiotherapy may allow for the skin and subcutaneous tissue sparing. These new selective RT techniques seem more appropriate in case of the combined BRAFi–RT treatment. Moreover, highly selective RT techniques such as intensity-modulated radiotherapy or stereotactic RT allow for hypofractionation, that is, reduction in the number of fractions and an increase in the fraction size. Consequently, hypofractionation allows administering RT in shorter overall treatment time, thus facilitating the combination of BRAFi and RT.

In a large multicentre analysis carried out to define the safe use of concomitant BRAFi and RT, it was highlighted that vemurafenib is a more potent radiosensitizer than dabrafenib and RT technique may influence the acute toxicity. In fact, no increased skin toxicity was reported after stereotactic RT (19 treated lesions), while acute radiodermatitis > Grade 2 was experienced by 46% of patients (p < 0.001) who received 3D conformal RT using a conventionally fractionated schedule. These data were BRAFi independent, even though vemurafenib was administrated to the majority of patients.^13^

In a recent study by Ly et al,^14^ where severe acute toxicity was documented in terms of increased haemorrhage risk (with no reported skin toxicity), the final recommendation was discontinuation of BRAFi for 1–2 weeks, both before and after stereotactic RT. This interruption did not worsen the control of systemic disease.

Moreover, few case reports have already been published focusing on skin toxicity related to the different radiation techinques. In a retrospective analysis of 12 patients (five treated with whole/partial brain RT; one treated with whole-brain RT and stereotactic RT as a boost; and six treated with stereotactic RT), no toxicities were reported except for brain necrosis in one patient.^15^ In a recently reported small series of five patients who underwent whole-brain RT (three patients) and stereotactic RT (two patients) concomitantly to BRAFi, there was no evidence of increased radiation-related toxicity, although an easily manageable radiation dermatitis (Grade 2 and 1) occurred in patients treated with the whole-brain approach.^8^

Another recent study by Gaudy-Marqueste^16^ was conducted to evaluate the feasibility of combined stereotactic radiosurgery (using a Gamma Knife system) with BRAFi in melanoma patients with brain metastases. Also in this series (30 patients), no scalp radiation dermatitis occurred during or after the radiation treatment.^16^

Our experience suggests, according to the previously mentioned literature data, that different doses to the cutaneous and subcutaneous tissues might influence the RT-related skin toxicity. The case described here, in fact, was treated at the same time with two different 3D conformal RT techniques that led to different distribution of the high doses into the cutaneous and subcutaneous tissues. The clinical evidence of different skin toxicity clearly demonstrated that this different dose distribution may influence the reaction of skin cells. To the best of our knowledge, this is the first such case but further prospective studies are required to confirm this finding. The use of the RT techniques correlating with the reduced skin toxicity might be indicated when concomitant RT and BRAFi is administrated. Such an approach could have a clinical impact on the management of patients with metastatic melanoma because it could permit the safe continuation of systemic treatment during the RT course. For this reason, radiation oncologists should carefully evaluate the RT technique when RT is used in combination with BRAFi and MEKi, although more evidence is still needed. This evidence should emerge from controlled clinical trials.

## Learning points

1. The association of BRAFi and RT could lead to severe skin toxicity.
2. Management of metastatic melanoma patients who have to be treated with RT and dabrafenib is still a hot topic, especially in deciding the interruption of systemic therapy during the course of RT.
3. The irradiation technique seems to influence skin toxicity when concomitant RT and BRAFi are administrated; this could be, in part, explained by the different dose distribution to the cutaneous and subcutaneous tissues.

## Consent

Written informed consent was obtained from the patient for publication of this case report and any accompanying images.
